# VEGF and bFGF induction by nitric oxide is associated with hyperbaric oxygen-induced angiogenesis and muscle regeneration

**DOI:** 10.1038/s41598-020-59615-x

**Published:** 2020-02-17

**Authors:** Naoki Yamamoto, Takuya Oyaizu, Mitsuhiro Enomoto, Masaki Horie, Masato Yuasa, Atsushi Okawa, Kazuyoshi Yagishita

**Affiliations:** 1 Department of Orthopaedic Surgery, Tokyo Medical and Dental University, Bunkyo-ku, Tokyo 113-8519 Japan; 2 Hyperbaric Medical Center, Tokyo Medical and Dental University, Tokyo, 113-8519 Japan; 3 Saiseikai Kawaguchi General Hospital, Kawaguchi-shi, Saitama 332-8558 Japan

**Keywords:** Biophysical chemistry, Blood proteins, Trauma, Orthopaedics

## Abstract

Hyperbaric oxygen (HBO) treatment promotes early recovery from muscle injury. Reactive oxygen species (ROS) upregulation is a key mechanism of HBO, which produces high O_2_ content in tissues through increased dissolution of oxygen at high pressure. Nitric oxide (NO), a type of ROS, generally stabilizes hypoxia-inducible factor (HIF) 1α and stimulates secretion of vascular endothelial growth factor (VEGF) and basic fibroblast growth factor (bFGF) from endothelial cells and macrophages, which then induces angiogenesis. The purpose of the present study was to investigate whether HBO could promote angiogenesis via induction of NO and induce muscle regeneration in contused rat skeletal muscles. The HBO protocol consisted of 2.5 atmospheres absolute (ATA) 100% oxygen for 120 minutes, once a day for 5 consecutive days. We also evaluated the effects of a ROS inhibitor (NAC) or NOS-specific inhibitor (L-NAME) on HBO. HBO significantly increased NO_3_^−^, VEGF, and bFGF levels and stabilized HIF1α within 1 day. HBO promoted blood vessel formation at 3–7 days and muscle healing at 5–7 days after contusion. Administration of both NAC and L-NAME before HBO suppressed angiogenesis and muscle regeneration even after HBO. HBO thus promoted angiogenesis and muscle regeneration mainly through generation of NO in the early phase after muscle contusion injury.

## Introduction

Muscle contusion injury is one of the common injuries in sports medicine^1^. Among muscle injuries, 90% are caused by contusions^2^ produced by high-energy blunt trauma from a non-penetrating object, or by excessive strain of the muscle^3^. Such injuries are usually treated non-operatively following the RICE (rest, ice, compression, and elevation) protocol and a short period of immobilization, followed by active and passive range-of-motion exercises^3^. In severe cases, muscle contusions cause vascular disruption^4,5^. Acute ischemia induces gradual deterioration of energy metabolism in muscle, followed by cell death or adaptation^6^. Delayed vascular repair can delay muscle regeneration, which may lead to increased fibrosis in skeletal muscle. It has been reported that decreased blood supply caused by delayed vascular repair delays the regeneration of soft tissue^7^. Thus, muscle regeneration, collateral formation, and angiogenesis are likely related, and blood vessel formation after injury is also essential for muscle regeneration^2,8^.

Early and efficient recovery of blood vessels may be important for recovery of motor function, especially with regard to muscle tensile strength, and may enable an early return to sports for athletes. Thus, early and appropriate treatment strategies for severe muscle injury focusing on angiogenesis are required.

After injury, among the factors associated angiogenesis, upregulation of VEGF^9–11^, basic fibroblast growth factor (bFGF)^6,11^, hepatocyte growth factor (HGF)^6^, and angiopoietin 2^11^ activates migration and proliferation of endothelial cells, and promotes angiogenesis^12,13^ Moreover, it has been reported that stabilization of hypoxia-inducible factor (HIF) 1α stimulates vascular endothelial growth factor (VEGF) secretion^11,12,14^ and is thus an important factor for VEGF-mediated angiogenesis. Restoration of VEGF levels has been shown to contribute to the dynamic process of capillary formation and muscle regeneration after muscle injury^15^. Skeletal muscles with increased vascularity have better regeneration than muscles with low vascularity^15^.

Hyperbaric oxygen treatment (HBO) promotes angiogenesis. HBO is a non-invasive treatment involving inhalation of pure oxygen for 60 to 90 minutes under 2 to 2.8 atmospheres of absolute pressure^14^. HBO increases the amount of dissolved oxygen in the blood, thus providing a reservoir of oxygen at the cellular level. The oxygen is carried not only by blood, but also by diffusion from the interstitial tissue, where a high concentration of oxygen is reached^6,11,14,16^. Thus, HBO improves oxygen delivery to areas with diminished blood flow. HBO temporarily increases levels of reactive oxygen species (ROS), mainly composed of superoxide (O_2_^−^), hydrogen peroxide (H_2_O_2_), nitric oxide (NO), and peroxynitrite (ONOO-)^17–20^. Continuous elevation of ROS is known as oxidative stress. However, transient elevation of ROS induces signal transduction cascades for a variety of growth factors, cytokines, and hormones^17–22^. These changes stimulate collagen synthesis^23^, proliferation of cells such as satellite cells^24–26^, and angiogenesis^9,27^.

HBO has been reported as an additional treatment for skeletal muscle injury, as HBO is clinically considered to promote muscle regeneration^28,29^ and accelerate the return to competition^30^. Our previous study showed that HBO accelerated the recovery of intact muscle volume, stimulated satellite cell proliferation, and promoted muscle regeneration via macrophage recruitment^31,32^. In a previous study, HBO increased mRNA levels of bFGF and HGF without upregulation of VEGF, increasing and promoted angiogenesis and muscle regeneration after ischemic muscle injury^6^. Although these reports indicate that blood vessel formation induced by angiogenic growth factors is crucial for regeneration after skeletal muscle injury, the particular changes in the expression of these angiogenic growth factors remain unclear.

We hypothesized that HBO increases ROS and NO levels and subsequently induces secretion of bFGF, HGF, and VEGF to accelerate revascularization, thereby supporting muscle regeneration. In this study, we investigated the effects of HBO on angiogenesis and muscle healing in a rat skeletal muscle contusion model. Furthermore, we assessed the effects of ROS and NO inhibitors to determine the mechanism underlying the effects of HBO.

## Results

### HBO enhanced angiogenesis in contused muscle

**Figure 1:**
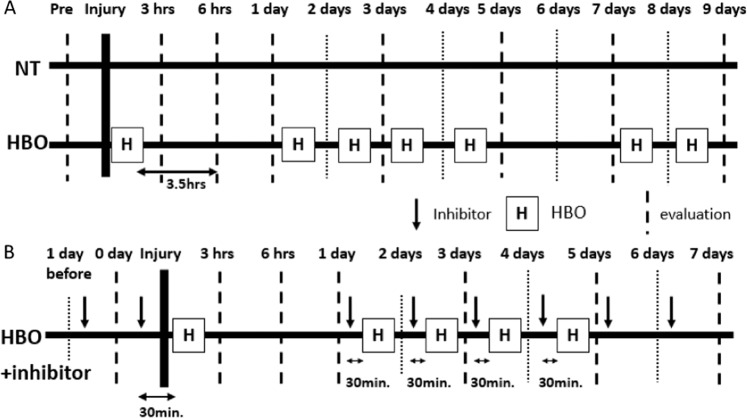
Treatment and evaluation schedule. (**A**) We performed HBO immediately after injury. Evaluation was performed at 3 hours, 6 hours, 1 day, 3 days, 5 days, 7 days, and 9 days after muscle contusion injury. (**B**) Inhibition schedule. ROS and NOS inhibitors were injected every day from one day before contusion injury to the day before measurement. Inhibitors were injected 30 minutes before administration of HBO.

**Figure 2:**
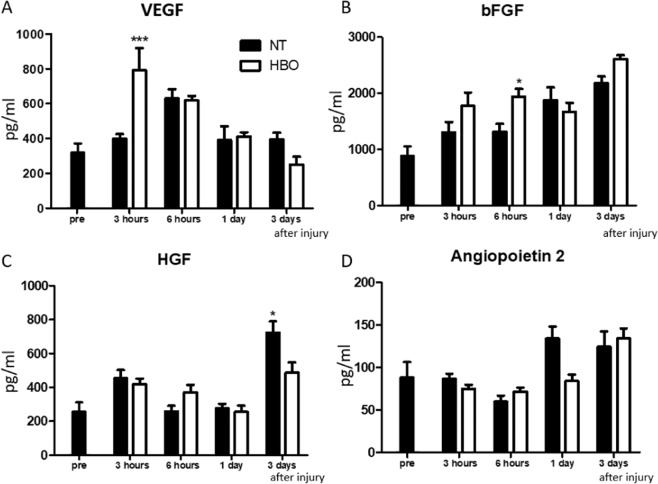
HBO promoted VEGF and bFGF synthesis but did not increase HGF and angiopoietin2 production after muscle contusion injury. (**A**,**B**) The amount of VEGF and bFGF protein in the injured gastrocnemius muscle was significantly increased at 3 or 6 hours after injury in the HBO group. (**C**,**D**) There was no significant difference in the amount of HGF and angiopoietin 2 protein at each phase in the HBO group, n = 6, using total 60 animals ***P < 0.001, *P < 0.05 using multiple-way ANOVA followed by Bonferroni post-tests. Data are the mean ± SEM.

We quantified the amounts of VEGF, bFGF, HGF, and angiopoietin 2 protein in contused muscles at 3 hours, 6 hours, 1 day, and 3 days after injury by ELISA (Fig. 1). VEGF levels were significantly increased in the HBO group at 3 hours after injury (NT group: 311.2 ± 58.2 pg/ml, HBO group: 827.5 ± 83.8 pg/ml, F(3, 20) = 21.21, p < 0.001.) (Fig. 2A). bFGF levels significantly increased in the HBO group at 6 hours after injury(NT group: 1314.6 ± 144.3 pg/ml, HBO group: 1939.8 ± 138.0 pg/ml, F(3,20) = 4.471, p = 0.03) (Fig. 2B). The amounts of HGF and angiopoietin 2 protein did not change in the HBO group (Fig. 2C,D).

**Figure 3:**
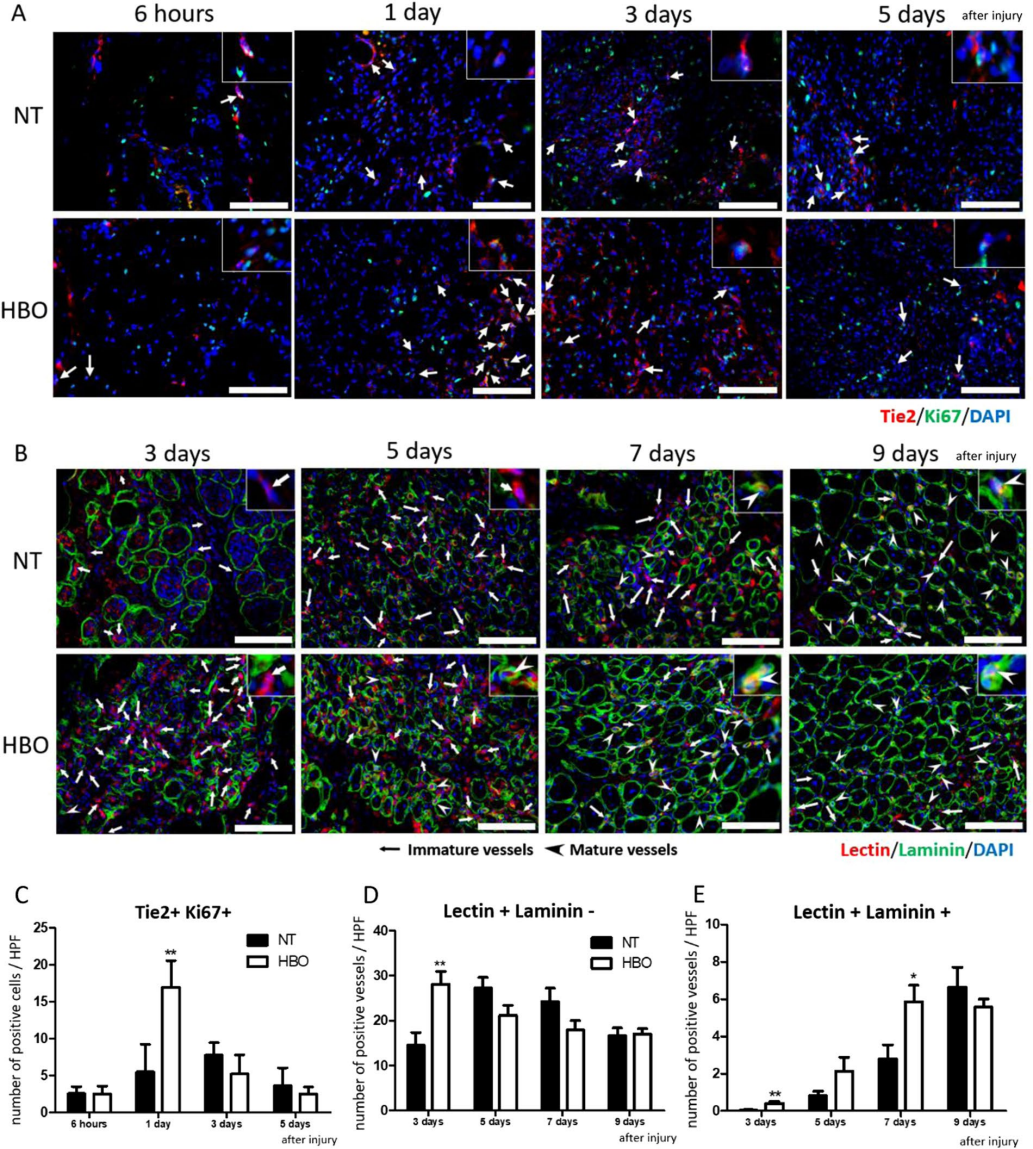
HBO accelerated proliferation of endothelial cells and increased the amount of both immature and mature blood vessels. (**A**) Representative images of endothelial cells positive for Tie2 (red), Ki67 (green), and DAPI (blue) in injured muscle (arrows). Scale bar: 100 µm. One of the double-positive cells is enlarged in the upper right of each image. (**B**) Representative images of immature blood vessels (inset) positive for tomato lectin (red) and DAPI (blue) (arrows), and mature vessels (inset) positive for tomato lectin, laminin (green), and DAPI (arrow heads) in injured muscle. Scale bar: 100 µm. One of the immature or mature vessels is enlarged in the upper right of each image. (**C**) The number of proliferating endothelial cells was significantly increased at 1 day after injury in the HBO group, n = 5, using total 65 animals **P < 0.01, using multiple-way ANOVA followed by Bonferroni post-tests. (**D**) The number of immature blood vessels was significantly increased at 3 days after injury in the HBO group, n = 5. **P < 0.01 using multiple-way ANOVA followed by Bonferroni post-tests. (**E**) The number of mature blood vessels in the HBO group was considerably increased at 3, 5, and 7 days after injury, n = 5, using 20 animals **P < 0.01, *P < 0.05 using multiple-way ANOVA followed by Bonferroni post-tests. Data are the mean ± SEM.

Next, we analyzed the number of proliferating endothelial cells in the contused muscles. Tie2 and Ki67 double-positive cells, representing endothelial cells and cells not in the quiescent phase, were counted at 6 hours and 1, 3, and 5 days after injury (Fig. 3A,C). Quantitative analysis showed a significant increase at 1 day in the HBO group (NT group: 10.7 ± 5.7 cells/HPF, HBO group: 29.9 ± 7.5 cells/HPF, F(1,8) = 20.500, p = 0.001).

Finally, we counted the number of immature and mature vessels (Fig. 3B,D,E). Generally, vessels are evaluated using vessel-specific tomato lectin, as immature vessels are stained with lectin and do not contain laminin. Mature vessels are stained with lectin and surrounded by laminin (Fig. 3B). Quantitative analysis showed a significant increase of immature vessels in the HBO group at 3 days after injury (3 days- NT group: 14.6 ± 2.8 vessels/HPF, HBO group: 28.1 ± 2.8 vessels/HPF, F(1,8) = 11.539, p = 0.009) (Fig. 3D). Moreover, the number of mature vessels in the HBO group was significantly increased at 3 and 7 days after injury (3 days- NT group: 0.04 ± 0.02 vessels/HPF, HBO group: 0.4 ± 0.1 vessels/HPF, F(1,8) = 12.226, p = 0.008; 7 days- NT group: 2.8 ± 0.8 vessels/HPF, HBO group: 5.9 ± 0.9 vessels/HPF, F(1,8) = 6.979, p = 0.03) (Fig. 3E). Overall, VEGF and bFGF levels were increased after HBO within 6 hours, and vascular formation based on the increase of endothelial cell proliferation occurred earlier in the contused muscles of the HBO group than in those of the NT group.

### HBO induction of NO mediated HIF1α stabilization and the effects of angiogenic factors in the contused muscle

**Figure 4:**
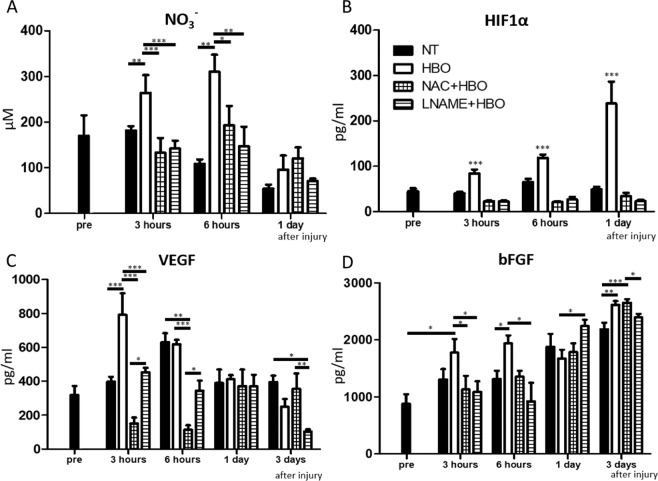
HBO-mediated upregulation of NO, HIF1α, VEGF, and bFGF was suppressed by NAC and L-NAME. (**A**) The amount of NO_3_^−^, the final oxidized product of NO, was significantly increased at 3 and 6 hours after injury in the HBO group, n = 6. ***P < 0.001, **P < 0.01, *P < 0.05 using multiple-way ANOVA followed by Bonferroni post-tests. Data are the mean ± SEM. (**B**) The amount of HIF1α protein was significantly increased at 3 and 6 hours and 1 day after injury in the HBO group, and this effect was suppressed under administration of both inhibitors, n = 6. ***P < 0.001, using multiple-way ANOVA followed by Bonferroni post-tests. (**C**,**D**) With NAC or L-NAME administration, VEGF and bFGF upregulation induced by HBO was suppressed at 3 or 6 hours after injury, n = 6, added total 48 animals ***P < 0.001, **P < 0.01, *P < 0.05 using multiple-way ANOVA followed by Bonferroni post-tests. Data are the mean ± SEM.

We hypothesized that upregulation of NO underlies the therapeutics effects of HBO. Because direct quantitation of NO production is difficult given its extremely short half-life, we measured the amount of NO_3_^−^, the final oxidized product of NO. We also administered an ROS inhibitor (N-acetylcysteine; NAC) or NO synthetase (NOS) inhibitor (nitro-L-arginine methyl ester; L-NAME) to investigate whether ROS or NO is required for the angiogenesis and muscle regeneration mediated by HBO. NO_3_^−^ production was significantly increased at 3 and 6 hours after injury in the HBO group (3 hours- NT group: 182.0 ± 9.1 µM, HBO group: 263.9 ± 39.4 µM, F(1,10) = 17.39, p = 0.0019; 6 hours -NT group: 108.9 ± 9.2 µM, HBO group: 310.1 ± 37.7 µM, F(1,10) = 26.85, p = 0.0025), and suppressed in the inhibitor + HBO group compared to the HBO group (3 hours- NAC + HBO group: 133.1 ± 32.0 µM, p = 0.0025 L-NAME + HBO group: 142.0 ± 17.5 µM, F(3,20) = 21.21, p < 0.001; 6 hours - NAC + HBO group: 199.2 ± 50.2 µM, p = 0.107, L-NAME + HBO group: 184.4 ± 42.6 µM, F(3,20) = 7.405, p = 0.05) (Fig. 4A). Next, we measured HIF1α protein and mRNA levels, as ROS and NO stabilize HIF1α and upregulate angiogenic growth factors^11,12,14^. HIF1α production was significantly increased at 3 and 6 hours and 1 day after injury in the HBO group (3 hours- NT group: 40.2 ± 3.5 pg/ml, HBO group: 84.6 ± 8.3 pg/ml, F(1,10) = 24.12, p < 0.001; 6 hours- NT group: 65.3 ± 6.9 pg/ml, HBO group: 118.6 ± 7.1 pg/ml, F(1,10) = 29.04, p < 0.001; 1 day- NT group: 49.2 ± 5.6 pg/ml, HBO group: 238.1 ± 48.3 pg/ml, F(1,10) = 15.07, p = 0.011), and was suppressed in the inhibitor + HBO group compared to the HBO group (3 hours- NAC + HBO group: 22.9 ± 1.5 pg/ml, p < 0.001, L-NAME + HBO group: 22.5 ± 2.2 pg/ml, F(3,20) = 38.51, p < 0.001; 6 hours- NAC + HBO group: 21.0 ± 1.7 pg/ml, p < 0.001, L-NAME + HBO group: 26.8 ± 5.6 pg/ml, F(3,20) = 61.16, p < 0.001; 1 day- NAC + HBO group: 34.1 ± 7.5 pg/ml, p < 0.001, L-NAME + HBO group: 23.3 ± 2.8 pg/ml, F(3,20) = 17.05, p < 0.001) (Fig. 4B). HIF1α mRNA expression did not increase after injury (Supplement 1C). HBO increased HIF1α protein levels without increasing the mRNA levels. This result suggests that HBO stabilizes HIF1α and prevents its degradation.

We also evaluated the effect of the inhibitors on the HBO-mediated induction of VEGF and bFGF. The upregulation of VEGF by HBO was significantly suppressed in the NAC + HBO and L-NAME + HBO groups at 3 and 6 hours and 3 days after injury compared to the HBO group (3 hours- NAC + HBO group: 150.7 ± 35.8 pg/ml, p = 0.046, L-NAME + HBO group: 359.5 ± 65.4 pg/ml, F(3,20) = 21.21, p = 0.0015; 6 hours- NAC + HBO group: 114.0 ± 26.0 pg/ml, p = 0.0085, L-NAME + HBO group: 343.4 ± 44.3 pg/ml, F(3,20) = 7.405, p = 0.12; 3 days- NAC + HBO group: 355.5 ± 90.6 pg/ml, p = 0.77, L-NAME + HBO group: 127.4 ± 16.5 pg/ml, F(3,20) = 4.144, p = 0.046) (Fig. 4C). The upregulation of bFGF by HBO was significantly suppressed in the NAC + HBO and L-NAME + HBO groups at 6 hours after injury compared to the HBO group (NAC + HBO group: 1356.3 ± 102.4 pg/ml, L-NAME + HBO group: 922 ± 326.9 pg/ml, F(3,20) = 4.471, p = 0.0015.) (Fig. 4D).

### ROS and NOS inhibition suppressed HBO-mediated angiogenesis and muscle healing

**Figure 5:**
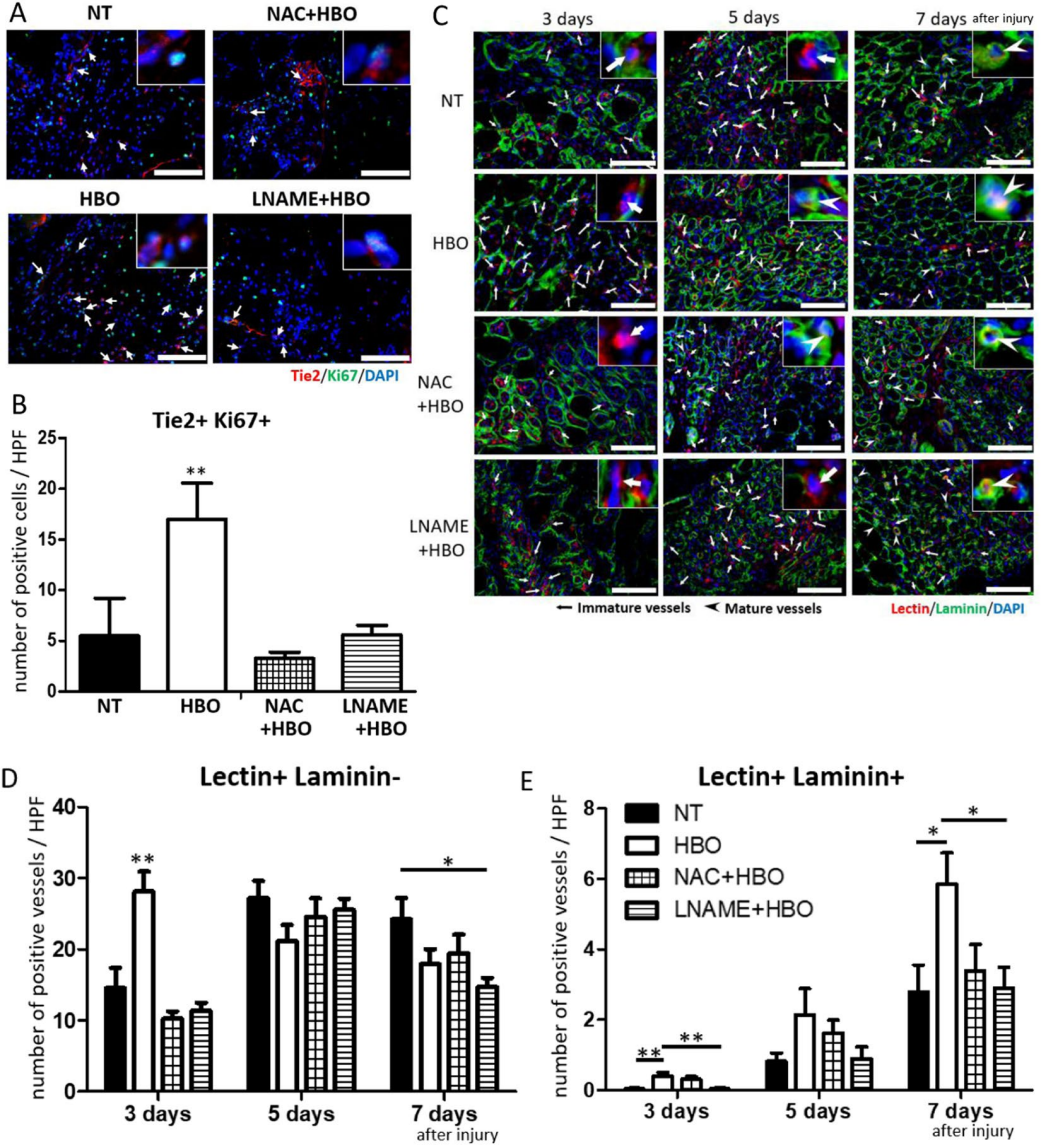
Effects of HBO on angiogenesis were suppressed by NAC or L-NAME. (**A**) Representative image of endothelial cells positive for Tie2 (red), Ki67 (green), and DAPI (blue) in injured muscle at 1 day after injury (arrows). Scale bar: 100 µm. One of the double-positive cells is enlarged in the upper right of each image. (**B**) Administration of NAC or L-NAME reduced the number of Tie2 and Ki67 double-positive cells at 1 day after injury in the HBO group. n = 5, added total 10 animals. **P < 0.01, using one-way ANOVA followed by Bonferroni post-tests. Data are the mean ± SEM. (**C**) Representative image of immature blood vessels positive for tomato lectin (red) and DAPI (blue), and mature vessels positive for tomato lectin, laminin (green), and DAPI in the injured muscle. Scale bar: 100 µm. One of the immature and/or mature vessels is enlarged on the upper right at each image. (**D**) The number of immature blood vessels in the NAC + HBO and LNAME + HBO groups was decreased at 3 days after injury. n = 5. (**E**) The number of mature blood vessels in the NAC + HBO and LNAME + HBO groups was decreased at 3, 5, and 7 days after injury. n = 5, added total 30 animals. **P < 0.01, *P < 0.05, using multiple-way ANOVA followed by Bonferroni post-tests. Data are the mean ± SEM.

Tie2 and Ki67 double-positive cells were less frequently observed in the NAC + HBO, L-NAME + HBO, and NT groups than in the HBO group (Fig. 5A). The number of Tie2 and Ki67 double-positive cells in the NAC + HBO and L-NAME + HBO groups was equal to that in the NT group (NAC + HBO group: 3.3 ± 0.6 cells/HPF, p = 0.0015, L-NAME + HBO group: 5.6 ± 0.9 cells/HPF, F(3,16) = 22.97, p < 0.001) at 1 day after injury (Fig. 5B).

The number of lectin+/laminin- vessels (immature vessels) was decreased significantly at 3 days after injury in the NAC + HBO and L-NAME + HBO groups (NAC + HBO group: 10.2 ± 1.1 cells/HPF, p < 0.001, L-NAME + HBO group: 11.3 ± 1.2 cells/HPF, F(3,16) = 14.74, p < 0.001) (Fig. 5D). The number of lectin + /laminin + vessels (mature vessels) at 3 and 7 days after injury was decreased in the L-NAME + HBO group (3 days- 0.04 ± 0.024 /HPF, F(3,16) = 7.407, p = 0.002; 7 days- L-NAME + HBO group: 2.9 ± 0.6 /HPF, F(3,16) = 3.565, p = 0.024) (Fig. 5E).

**Figure 6:**
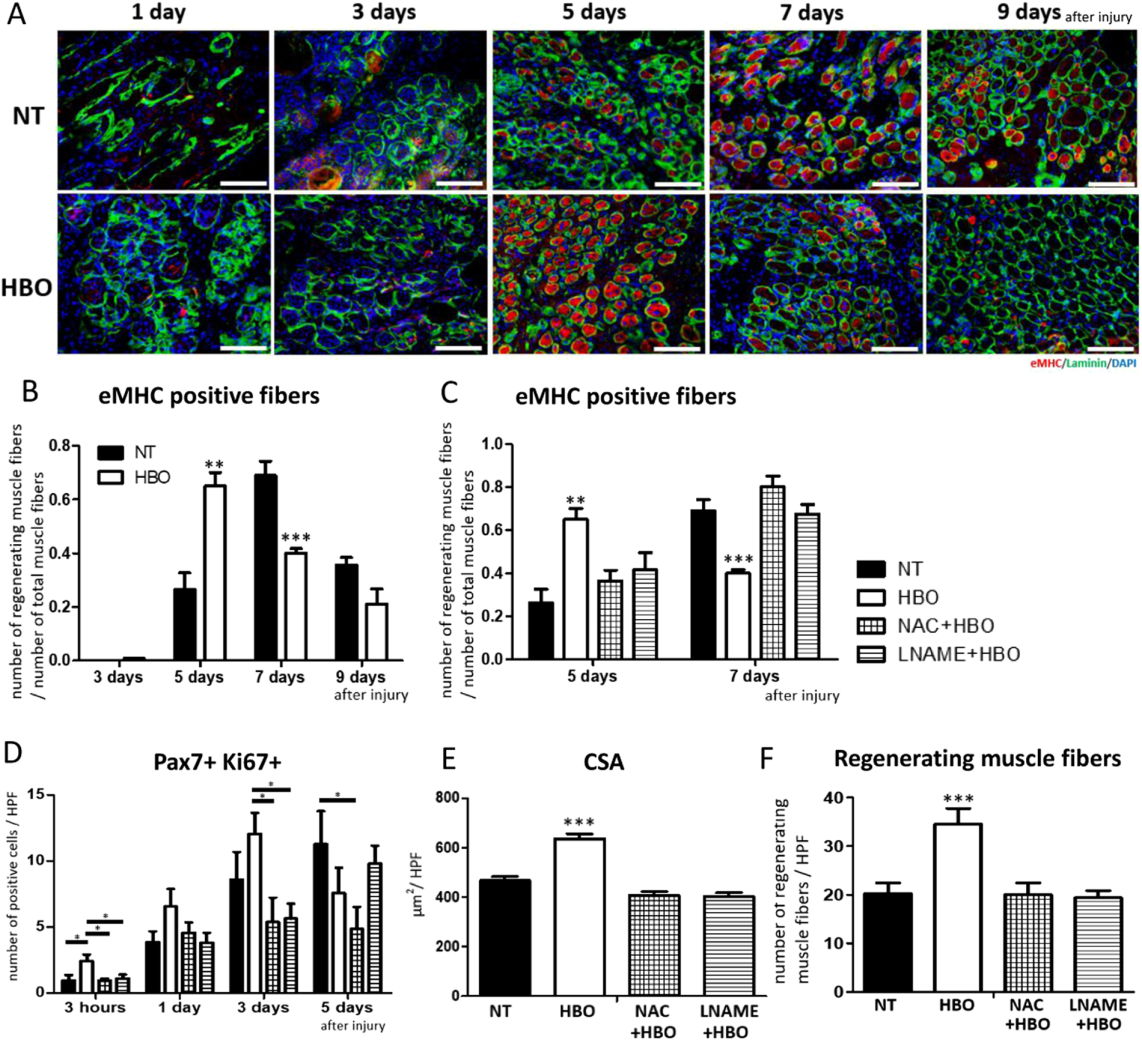
HBO-mediated muscle regeneration was also suppressed by NAC and L-NAME inhibition after muscle contusion injury. (**A**) Representative image of regenerating muscle fibers stained for eMHC and laminin at 1, 3, 5, 7, and 9 days after injury. Scale bar: 100 µm. (**B**) Relative number of eMHC- and laminin- double-positive fibers in the HBO group was significantly increased at 5 days after injury, and decreased at 7 days, n = 5, using 20 animals. ***P < 0.001, **P < 0.01, using multiple-way ANOVA followed by Bonferroni post-tests. Data are the mean ± SEM. (**C**) Under inhibition by NAC or L-NAME, relative number of eMHC- and laminin- double-positive fibers were almost equal to those in the NT group, regardless of whether HBO was performed, n = 5, ***P < 0.001, **P < 0.01, using multiple-way ANOVA followed by Bonferroni post-tests. (**D**) Under inhibition by NAC or L-NAME, the number of Pax7- and Ki67-double-positive cells was significantly suppressed compared to that in the HBO group, n = 5, *P < 0.05, using multiple-way ANOVA followed by Bonferroni post-tests. (**E**,**F**) Under inhibition by NAC or L-NAME, CSA and the number of regenerating muscle fibers in the HBO group were suppressed at 5 days after injury. n = 5. ***P < 0.001, using one-way ANOVA followed by Bonferroni post-tests. Data are the mean ± SEM.

We then evaluated muscle regeneration in the inhibitor + HBO groups. We investigated embryonic myosin heavy chain (eMHC) and laminin double-positive fibers as regenerating muscle fibers. Fibers positive for laminin but not eMHC were considered mature muscle fibers. We calculated the ratio of the number of immature to that of mature fibers at 3, 5, 7, and 9 days after injury (Figs. 6A,B). In the HBO group, the ratio was significantly increased at 5 days and decreased at 7 days after injury (5 days- NT group: 0.265 ± 0.063 ratio/HPF, HBO group: 0.651 ± 0.050 ratio/HPF, F(1,8) = 22.99, p = 0.0016, 7 days- NT group: 0.690 ± 0.053 ratio/ HPF, HBO group: 0.402 ± 0.016 ratio/HPF, F(1,8) = 27.02, p = 0.0041) (Fig. 6B). However, the ratio of regenerating to mature muscle fibers in the inhibitor + HBO groups was similar to that in the NT group regardless of HBO induction (5 days- NAC + HBO group: 0.367 ± 0.049 ratio/HPF, p = 0.239, L-NAME + HBO group: 0.417 ± 0.081 ratio/HPF, F(3,16) = 6.89, p = 0.179; 7 days- NAC + HBO group: 0.794 ± 0.102 ratio/HPF, p = 0.160, L-NAME + HBO group: 0.643 ± 0.090 ratio/HPF, F(3,16) = 15.66, p = 0.850) (Fig. 6C). The inhibitors diminished the effect of HBO on muscle regeneration. Next, to evaluate satellite cell proliferation, we counted Pax7 and Ki67 double-positive cells (representative of satellite and non-quiescent cells, respectively) at 3 hours and 1, 3, and 5 days after injury. The number of Pax7+/Ki67 + cells was increased in the HBO group at 3 hours, but did not increase at 3 hours after injury in the NAC + HBO and L-NAME + HBO groups compared to the HBO group (3 hours- NT group: 0.96 ± 0.41 cells/HPF, p = 0.05, HBO group: 2.4 ± 0.49 cells / HPF, NAC + HBO group: 0.95 ± 0.17 cells/HPF, p = 0.044, L-NAME + HBO group: 1.1 ± 0.29 cells/HPF, F(3,16) = 3.837, p = 0.097) (Fig. 6D, Supplement 2A). We stained sections with H&E and evaluated the regenerating muscle fibers with a central nucleus by measuring their cross-sectional area (CSA) and counting them at 5 days after injury (Figs. 6E,F). In the HBO group, the CSA of the regenerating muscle fibers was higher than that in the NT, NAC + HBO, and L-NAME + HBO groups at 5 days after injury (NT group: 466.0 ± 17.3 μm^2^/HPF, HBO group: 635.8 ± 19.7 μm^2^/HPF, NAC + HBO group: 407.1 ± 15.9 μm^2^/HPF, LNAME + HBO group: 402.5 ± 16.0 μm^2^/HPF, F(3,16) = 20.7, p < 0.001) (Fig. 6E). The number of regenerating muscle fibers was significantly increased in the HBO group compared to the NT group. However, there was no significant increase in the NAC + HBO and L-NAME + HBO groups at 5 days after injury (NT group: 20.22 ± 2.2 fibers/HPF, HBO group: 34.6 ± 3.2 fibers/HPF, NAC + HBO group: 20.0 ± 2.4 fibers/HPF, p = 0.0031, LNAME + HBO group: 19.4 ± 1.5 fibers/HPF, F(3,16) = 9.261, p = 0.0021) (Fig. 6F).

**Figure 7:**
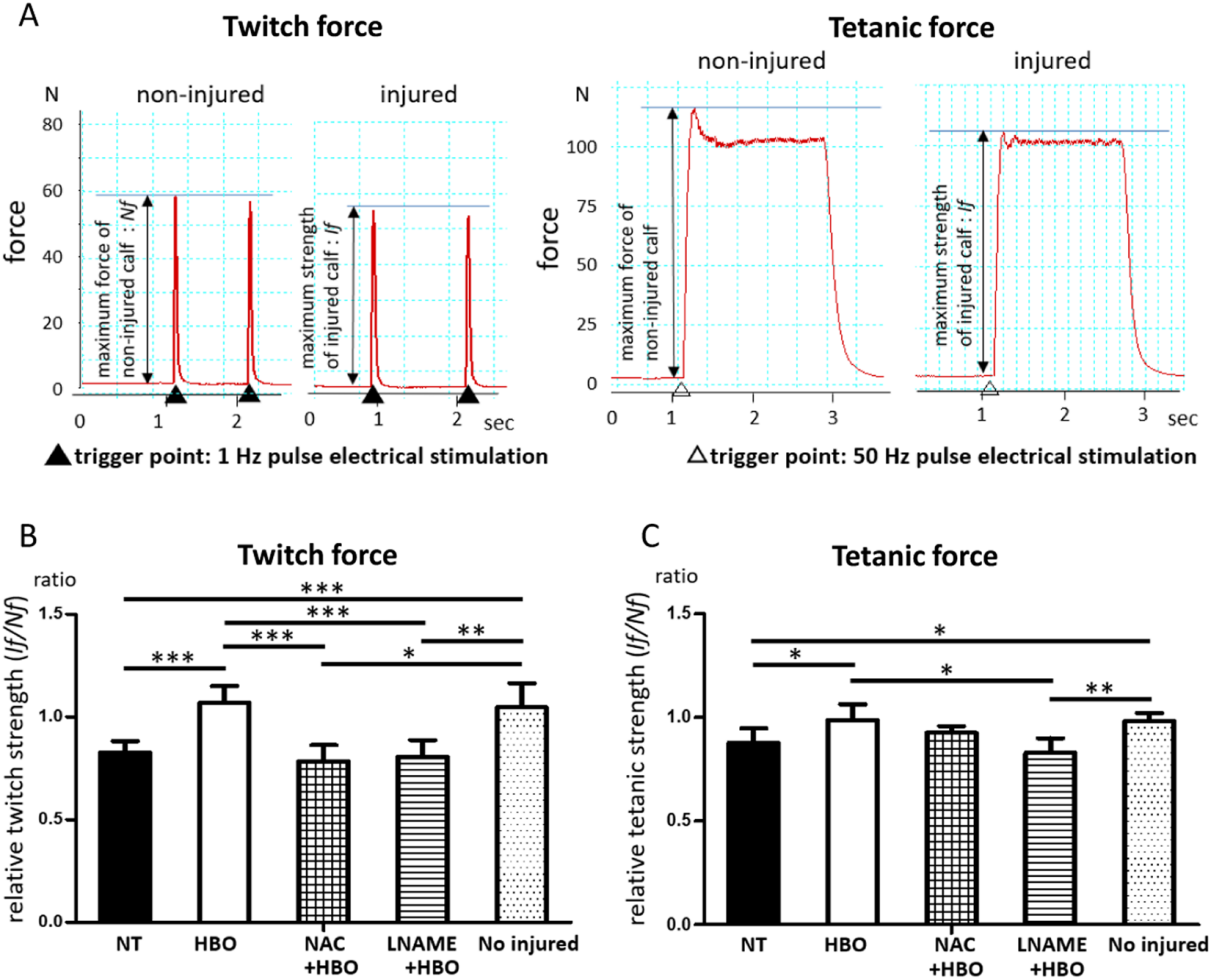
HBO recovered muscle tensile strength, and this effects was diminished by NAC and L-NAME. (**A**) Twitch and tetanic forces were recorded in the NT group at 7 days after injury, and subsequently the ratios of injured leg (If) to non-injured leg forces (Nf) were calculated. (**B**,**C**) The ratio of twitch and tetanic forces in the injured leg relative to those in the non-injured leg was calculated at 7 days after injury. Administration of NAC or LNAME eliminated the increase in relative twitch force and tetanic force in the HBO group, n = 6 using 30 animals. ***P < 0.001, **P < 0.01, *P < 0.05, using one-way ANOVA followed by Bonferroni post-tests. Data are the mean ± SEM.

Finally, we measured the muscle tensile isometric strength, twitch force, and tetanic force at 7 days after injury (Fig. 7A–C). In the HBO group, the twitch force and tetanic force were significantly increased. The HBO-induced increase in twitch force was diminished in the NAC + HBO and L-NAME + HBO groups (NT group: 0.83 ± 0.1, HBO group: 1.06 ± 0.08, NAC + HBO group: 0.78 ± 0.08, L-NAME + HBO group: 0.81 ± 0.1, F(4,25) = 12.77, p < 0.001) (Fig. 7B). The HBO-induced increase in tetanic force was also diminished in the L-NAME + HBO group (NT group: 0.87 ± 0.07, HBO group: 0.96 ± 0.08, NAC + HBO group: 0.92 ± 0.03, L-NAME + HBO group: 0.88 ± 0.12, F(4,25) = 0.671, p = 0.011) (Fig. 7C).

## Discussion

In this study, we demonstrated that HBO increased VEGF and bFGF levels within 6 hours after contusion injury. After HBO, the concentration of NO in the injured muscle was increased, and consequently HIF1α was stabilized. After these changes occurred, endothelial cells proliferated and blood vessel formation was accelerated, resulting in increased regeneration of muscle fibers and muscle strength. We also investigated whether the regenerative effects of HBO on angiogenesis and muscle regeneration could be attributed to ROS and NO by administering the inhibitors NAC and LNAME.

Skeletal muscle healing after injury is characterized by degeneration, inflammation, and regeneration stages^2,33^. Each of these stages entails a complex sequence of physiological and cellular events, which are considered to depend on an adequate blood supply for transport of cells and metabolites^7^. After severe muscle damage, the vasculature is largely destroyed^2,29^.

Angiogenesis generally occurs in response to expression of HIF1α and VEGF that starts within 6 hours after injury^7^, and upregulation of bFGF, HGF, and angiopoietin 2 from 1 day^6,34^. Endothelial cells are activated and proliferate at 3 days^35,36^, and the peak of angiogenesis with vessel formation occurs at 5–14 days^13,37^. These results in previous reports were consistent with what we observed here in the NT group. In contrast, in the HBO group, the increase of HIF1α, and VEGF production occurred at 3 hours, peak bFGF production was at 6 hours, peak endothelial cell activation was at 1 day, immature vessels were observed at 3 days, and mature vessel formation occurred at 5–7 days after injury. The number of regenerating muscle fibers was increased at 5–7 days, and muscle strength was improved at 7 days. However, HBO did not promote an increase of HGF and angiopoietin 2 levels after injury. These results suggest that HBO promotes VEGF- and bFGF-induced blood vessel regeneration, which results in muscle healing.

We also investigated the effects of ROS and NO inhibition on HBO-mediated muscle regeneration. ROS without NO are produced by mitochondria from nicotinamide adenine dinucleotide phosphate (NADPH)^17–22^. In contrast, NO is produced by NOS in endothelial cells and macrophages^38^. NAC is a precursor of glutathione with a thiol group maintained in a reduced state and good intracellular penetration properties; the thiol group has antioxidant effects for all ROS^39^. L-NAME inhibits all NOS non-selectively by competing with arginine for the active site of NOS, which produces NO^25,40^. As the half-life of NO is relatively short, we measured NO_3_^−^, the final oxidized product. In this study, we measured the time course of NO_3_^−^ levels and found a significant increase of NO_3_^−^ in the HBO group. Inhibition by NAC and L-NAME before HBO treatment diminished the increase of NO_3_^−^ and the effects of HBO on HIF1α, VEGF, and bFGF levels as well as endothelial cell proliferation, angiogenesis, satellite cell proliferation, muscle fiber regeneration, and muscle tensile strength. The upregulation of VEGF by HBO was suppressed by both inhibitors, and the inhibitory effect was stronger for NAC than for L-NAME. This indicates that VEGF upregulation by HBO was associated with both NO and ROS. The inhibitory effects of NAC and L-NAME were similar for the other factors, suggesting that NOs play a key role in the angiogenesis and muscle regeneration promoted by HBO^38,41^. In contrast, when NAC and L-NAME were administered without performing HBO after contusion injury, angiogenesis and muscle regeneration were not inhibited (Supplement 2B, supplement 3, 4), except for the inhibition of bFGF expression by NAC (Supplement 2C). It has reported that the normal upregulation of bFGF is reduced under NAC inhibition after injury^42,43^. Thus, it is considered that the inhibitors themselves did not affect vessel formation and muscle regeneration during the recovery from muscle injury. Interestingly, at 3 days after injury in the relatively late phase, the VEGF level was significantly decreased in both the L-NAME + HBO group and the L-NAME + NT group. Although NAC inhibits only iNOS, L-NAME inhibits all NOS, including eNOS, nNOS, and iNOS, strongly and non-selectively^44,45^ Inhibition of eNOS causes vascular endothelial cell disorders^46,47^. Thus, VEGF production may be decreased by endothelial cell damage caused by L-NAME-mediated eNOS inhibition.

In this study, we evaluated HIF1α, VEGF, bFGF, HGF, and angiopoietin as factors that promote angiogenesis in different ways. HIF1α stabilization stimulates production of VEGF^11,12,14^ and activates the VEGF-R2 receptor in endothelial cells. bFGF activates proliferation of endothelial cells via activation of the FGF receptor^48^. HGF promotes mitogenic and morphogenic functions of endothelial cells via activation of c-Met/HGF receptor^49^. Angiopoietin 2 activates the Tie2 receptor in endothelial cells^50^.

Here, HBO increased VEGF and bFGF levels within 6 hours after injury but did not increase the levels of HGF and angiopoietin 2. The increase in VEGF levels during HBO is considered to have occurred through two mechanisms. First, HBO provides oxygen for NO production in injured tissue by increasing oxygen partial pressure gradients between healthy and hypoxic tissues^51^. Second, HBO promotes the stabilization of HIF1 by increasing levels of NO, which decreases prolyl hydroxylase (PHD) activity. The subsequent stabilization of HIF1 induces VEGF expression^14,52^. In our study, mRNA levels of HIF1α did not increase, but the amount of HIF1α protein increased at 3 and 6 hours and 1 day after contusion injury. Under normoxic conditions, the mRNA expression of HIF1α is maintained, but HIF1α protein levels are kept low due to rapid degradation by PHD^14^. HBO stabilizes HIF1α by decreasing PHD activity^11,12,14^. The increase in HIF1α protein levels without a concomitant increase in mRNA levels suggests that HBO stabilized HIF1α in the contused skeletal muscle in the early phase after injury. Stabilization of HIF1α results in an increase in VEGF levels^11,12,14,51^. NO stimulates bFGF production in endothelial cells, which accelerates the proliferation of endothelial cells^53^. In the present study, HBO increased NO levels, which likely increased VEGF levels via HIF1α stabilization and bFGF levels via NO in the injured muscle.

After endothelial cells are stimulated by VEGF, the laminin-containing basement membrane is destroyed, and the endothelial cells start to proliferate abnormally at 5 days after injury^35,36^. Subsequently, the laminin is re-formed, and the abnormal proliferation of blood vessel cells ceases. The blood vessels then become mature, and angiogenesis is completed at 7 to 14 days^13,32^. In the HBO group in the present study, the number of proliferating endothelial cells was increased at 1 day after injury, and then the number of immature vessels increased at 3 days and decreased from 5 days after injury. The number of immature and mature blood vessels was almost equal to that in the NT group at 9 days after injury. HBO thus accelerates angiogenesis by stimulating endothelial cells but does not interfere with the total number of regenerated mature vessels.

Additionally, in the present study, HBO accelerated muscle healing from the early phase, and L-NAME and NAC administration suppressed this effect. We measured muscle healing in the group that received only 1 HBO treatment at 3 days after injury, i.e., at a later time point. This group did not show any difference compared to the NT group. Thus, later induction of HBO seems to be not effective (Supplement 5). Thus, HBO appears to act at early time points and accelerate physiological angiogenesis and muscle regeneration.

There are several limitations in the present study. First, we did not use an inhibitor that inhibits ROS without inhibiting NO. There are several NAPDH inhibitors that are selective for ROS without NO. However, these inhibitors also inhibit MAPK and COX pathways, which are also involved in angiogenesis. Thus, these inhibitors could not be used to evaluate the effects of ROS in the present study because of the confounding effects on angiogenesis. Instead, we used NAC, which inhibits all ROS, and L-NAME, which inhibits only NO. Although the effect of ROS can thus be determined by subtracting the effect of L-NAME from that of NAC, the measurement is still indirect. Additionally, the muscle contusion injury model in this study may not accurately represent most occupation- and sports-related muscle injuries. Even though the model that we used is not the same as human injury, it is widely adopted as a skeletal muscle injury model to study the muscle-healing process^34–36,54^. Furthermore, we did not investigate the most effective treatment condition for angiogenesis and muscle regeneration. Further studies that evaluate the most effective treatment protocol with regard to oxygen dose, pressure, timing, and frequency are needed.

In conclusion, HBO increased NO, VEGF, and bFGF levels and stabilized HIF1α, and subsequently accelerated endothelial and satellite cell proliferation and promoted angiogenesis and muscle regeneration after skeletal muscle contusion injury. This injury model is considered to simulate clinical skeletal muscle injury in terms of effects on not only skeletal muscle but also blood vessels. By using this injury model, we were able to evaluate both muscle regeneration and angiogenesis. Administration of NAC and L-NAME before HBO diminished the effects of HBO. The upregulation of NO is crucial for the effects of HBO on the muscle contusion injury. These findings provide basic evidence to support the clinical indication of HBO for skeletal muscle contusion injury.

## Materials and Methods

### Animals and contusion model

All animal experiments were performed under approved protocols and in accordance with the recommendations of the Institutional Animal Care and Use Committee of Tokyo Medical and Dental University. There is no conflict of interests in terms of the affiliations of researchers. Male 10-week-old Wistar rats weighing 250–300 g were kept in standard cages under a constant temperature and light/dark cycle of 12 hours each, with the light/dark hours changing automatically, and given water and food (MF; Oriental Yeast, Tokyo, Japan) ad libitum throughout the experimental period. The experiments were conducted mainly during the day from 6:00 AM to 6:00 PM, and the total number of rats used was 445 animals. The animals were anesthetized with an intraperitoneal injection of chloral hydrate (280 mg/kg) or gas anesthesia (1.5% to 3% isoflurane, 1.5 L/min flow). Muscle contusion was induced by the modified mass-drop method^55^ under gas anesthesia in the right calf muscle of the rats and subsequently in the left calf muscle to reduce the sample number of the rats. We used contralateral controls in the same animal to calculate the ratio of the strength of injured muscle to that of non-injured muscle when measuring muscle strength. First, the targeted hind limb was fixed distally at the Achilles’ tendon and proximally at the gastrocnemius muscle using percutaneous needles. The calf was placed on silicon clay (Therapy Putty; AliMed, Massachusetts, US). A solid aluminum cylinder (640 g) was dropped from a height of 250 mm onto the impactor (diameter 10 mm, hemispherical surface), which was placed on the belly of the medial calf of the rat. Subsequently, rats were randomly assigned to either no treatment (NT) or HBO treatment (HBO) after muscle contusion. If pain was apparent in the contused rats, we applied an analgesic agent such as felbinac gel.

### HBO protocol

Approximately 15 minutes after contusion injury, the rats in the HBO group were placed in a hyperbaric experimental chamber in which 100% oxygen was administered at 2.5 ATA pressure for 2 h, with 15 minutes for compression, 120 minutes of exposure at 2.5 ATA, and 15 minutes of decompression under 100% oxygen. The compression and decompression speeds were 0.1 ATA/min. In the clinic, HBO treatment is performed five times a week (except on weekends); thus, we also performed the same HBO protocol in this study, where HBO was performed once a day for 5 consecutive days (Fig. 1).

### Histological evaluation

We sacrificed all rats by overdose of isoflurane at each time point. When collecting the tissues, we separated the gastrocnemius and soleus muscles and collected the gastrocnemius, and froze the samples in liquid nitrogen-cooled 2-methylbutane. The muscles were stored at −80 °C until further analysis. Transverse sections of the calf muscles were cut at 20 µm using a cryostat (CM 300; Leica Japan, Tokyo, Japan) at 2, 4, 6, 8, 10, and 12 mm proximal from the end of the Achilles’ tendon, and stored at −30 °C. The sections were stained with hematoxylin and eosin (H&E). Images were obtained on a microscope (Olympus BX51; Olympus). In the evaluation of regenerated fibers, myofibers with centrally located nuclei were defined as regenerating fibers^31,32,56^. The injured area was determined as the cell-invaded area. We randomly selected 10 high-power fields (HPFs) from the injured area and counted the number of regenerating myofibers in the injured area. Moreover, we randomly selected 250 myofibers with regenerating myofibers and measured the cross-sectional areas (CSA) using the ImageJ software (ImageJ; National Institutes of Health, Bethesda, MD) (n = 5).

### Immunohistochemical analysis

Transverse sections of the gastrocnemius muscle were immersed in blocking solution (5% normal goat serum in PBS with 0.5% Triton X-100) for 30 minutes at 1, 3, 5, 7 and 9 days after injury, and then incubated overnight at 4 °C with the primary antibody (Tie2, mouse monoclonal antibody, BD Biosciences, Bedford, MA, USA, 1:100; Ki67, rabbit polyclonal antibody, Novus Biologicals, LCC, USA, 1:200; Pax7, mouse monoclonal antibody, R&D Systems, Minneapolis, MN, USA, 1:100; tomato lectin, mouse monoclonal antibody, Vector, Burlingame, CA, 1:200; laminin, rabbit polyclonal antibody, Sigma Aldrich, St. Louis, Mo, 1: 200; eMHC, mouse monoclonal antibody, Developmental Studies Hybridoma Bank, Iowa city, Iowa, USA, 1:200) diluted with PBS. The sections were washed 3 times for 5 minutes each with PBS and then incubated with secondary antibodies (goat anti-mouse IgG-Alexa Fluor 594, goat anti-rabbit IgG-Alexa Fluor 488; Life Technologies Japan, Tokyo, Japan) diluted 1:400 in PBS for 1 h. The sections were washed 3 times for 5 minutes with PBS. Finally, the sections were incubated for 1 min with DAPI (Life Technologies), washed with PBS, and mounted in mounting solution (PermaFluor; Thermo Fisher Scientific Japan, Yokohama, Japan). Positively stained cells were counted in 10 high-power fields (HPFs) using the histological procedures described above (n = 5).

### ELISA and nitrite measurement

Gastrocnemius muscles were collected from the hind limbs before injury, 3 and 6 hours after injury, and 1 and 3 days after injury. The samples were trimmed, frozen, and crushed using a cell crusher. The samples were homogenized with 1,000 µl of lysis/extraction reagent (CelLytick; Sigma-Aldrich, St. Louis, Mo.) and centrifuged at 14,000 rpm for 10 minutes at 4 °C, and the supernatant was then extracted for assay. The concentration of VEGF, bFGF, HGF, angiopoietin2, and HIF1 was measured using an enzyme-linked immunoassay (ELISA) kit (HIF1, Cell Biolabs, San Diego, USA; VEGF/bFGF/HGF/angiopoietin 2, R&D Systems, Minneapolis, MN) according to the manufacturer’s protocol (n = 6). The concentration of nitrite was measured using the OxiSelect Nitrite Assay Kit (Cell Biolabs, San Diego, USA) according to the provided protocol (n = 6).

### Quantitative reverse transcription polymerase chain reaction (qRT-PCR)

Gastrocnemius muscles were snap-frozen in liquid nitrogen and stored at −80 °C at each time point after the injury. Total RNA including the RNA fraction was isolated by lysis in 1 ml of Qiazol Total RNA Isolation Reagent (Qiagen, Hilden, Germany) using the RNeasy Mini Kit (Qiagen). One microgram of total RNA was reverse transcribed using the PrimeScript RT reagent Kit (TAKARA, Tokyo, Japan). Quantitative PCR was performed using GoTaq qPCR Master Mix (Promega), a qPCR System (Mx3000P; Agilent Technologies), 20 ng of cDNA, and specific primers according to the manufacturer’s instructions. The primer sequences were as follows: HIF1, 5′-GATTAGCCGAGTGCTCAGAATCAAG-3′ (forward) and 5′ = CAGGGCCAACCACTGTTTCATA-3′ (reverse); GAPDH, 5′-GGCACAGTCAAGGCTGAGAATG- 3′ (forward) and 5′- ATGGTGGTGAAGACG CCAGTA-3′ (reverse). The expression of the target mRNA was normalized to that of glyceraldehyde 3-phosphate dehydrogenase (GAPDH) mRNA. Relative quantification of gene expression was performed based on the comparative CT (threshold cycle value) method (ΔCT = CT gene of target - CT GAPDH gene). A comparison of gene expression in different samples was performed based on differences in the ΔCT of individual samples (ΔΔCT) (n = 4).

### ROS and NOS inhibitor administration

The ROS inhibitor N-acetyl cysteine (NAC, Wako, Tokyo, Japan) and NOS inhibitor N-nitro-L-arginine methyl ester hydrochloride (L-NAME, Sigma Aldrich, St. Louis, MO, USA) were peritoneally injected daily from the day before injury. Subsequently, rats were randomly divided into no treatment (NAC + NT, LNAME + NT) or HBO treatment (NAC + HBO, LNAME + HBO) groups after muscle contusion. On the day of HBO treatment, the inhibitor was administered at a dose of 0.018 mg/g 30 minutes before HBO as previously described^57^ (Fig. 1).

### Measurement of muscle tension isometric strength

Physiological testing was performed using a modified muscle tensile strength test at 7 days after contusion^58^. Rats were anesthetized intraperitoneally with chloral hydrate (280 mg/kg), placed on the platform in a prone position, and maintained at 37 °C using a heating pad. The Achilles tendon was sectioned 1 cm distal to the end of the muscle and sutured securely with 4–0 nylon using the Kessler method^59^. The exposed tendons and muscles were kept moist by periodically applying isotonic saline. A transducer (TB-653T; Nihon Koden, Tokyo, Japan), sensor interface (Power lab; AD Instruments Japan, Nagoya, Japan), and software (Power lab software; AD Instruments Japan) were used to measure the tension of the tendon. All data were displayed and stored on a computer using a custom-made program in LabView (National Instruments). General stimulation of the tibial nerve at 1 Hz (twitch) or 50 Hz (tetanus) was performed using surface electrodes (UL2–2020, Unique Medical Co., Ltd., Tokyo, Japan), and the maximum strength of Neurostimulus (Neuropack μ; Nihon Koden) was recorded^31,32^. The stimulation pulse was performed at the minimum voltage that caused the gastrocnemius muscle to reach its maximum contraction. The maximum crimp and tetanus isometric tensile strength of the injured (If) and non-injured (Nf) legs were measured. Before injury, there was no difference between the right and left sides (Supplement 1D). The strength ratio of the injured muscle to the non-injured muscle (ratio of If to Nf) was calculated (n = 6).

### Statistics

Data are presented as the mean ± standard error of the mean (SEM). All analyses were two-sided, with a significance level of 5%. Statistical analysis was performed using Windows SPSS version 23.0 (IBM Japan, Ltd., Tokyo, Japan). Data were analyzed for normality using the Shapiro-Wilk test and for homogeneity using the Levine test. We investigated longitudinal effects, the effect of HBO treatment, and the interaction between longitudinal effects and treatment using two-way ANOVA, with the number of proliferating endothelial cells, immature and mature vessels, proliferating satellite cells, muscle fibers stained with eMHC and laminin, amount of nitrite, relative expression ratio, and ELISA data under inhibition as the outcome means. The number of proliferating endothelial cells, number of regenerating muscle fibers with H&E staining, muscle tensile strength under inhibition, and CSA of regenerating muscle fibers with H&E staining under inhibition were compared across five or six groups (NT, HBO, NAC + HBO, LNAME + HBO, NAC + NT, LNAME + NT or NT, HBO, NAC + HBO, LNAME + HBO, non-injured) by one-way ANOVA followed by Bonferroni post-tests based on the normal distribution and homoscedasticity of the data. The twitch and tetanic force of the right and left gastrocnemius muscle before injury, which had only a normal distribution and without homoscedasticity, were subjected to Welch’s t-test for comparison between two groups.

## Supplementary information

Supplementary information.

## References

[1] Garrett WE (1996). Muscle strain injuries. Am. J. Sports Med..

[2] Smith C, Kruger MJ, Smith RM, Myburgh KH (2008). The Inflammatory Response to Skeletal Muscle Injury: Illuminating Complexities. Sports Med..

[3] Garrett WE (1990). Muscle strain injuries: clinical and basic aspects. Med. Sci. Sports Exerc..

[4] Beiner JM, Jokl P, Cholewicki J, Panjabi MM (1999). The effect of anabolic steroids and corticosteroids on healing of muscle contusion injury. Am. J. Sports Med..

[5] Nathan C, Cunningham-Bussel A (2013). Beyond oxidative stress: an immunologist’s guide to reactive oxygen species. Nat. Rev. Immunol..

[6] Asano T (2007). Hyperbaric Oxygen Induces Basic Fibroblast Growth Factor and Hepatocyte Growth Factor Expression, and Enhances Blood Perfusion and Muscle Regeneration in Mouse Ischemic Hind Limbs. Circ. J..

[7] Bray RC, Leonard CA, Salo PT (2003). Correlation of healing capacity with vascular response in the anterior cruciate and medial collateral ligaments of the rabbit. J. Orthop. Res..

[8] Oscar O (2007). Delayed angiogenesis and VEGF production in CCR2−/− mice during impaired skeletal muscle regeneration. Am. J. Physiol. Regul. Integr. Comp. Physiol..

[9] Malgorzata M, Olga H, Margaret DB, Haley S (2005). Nitric oxide, VEGF, and VEGFR-2: interactions in activity-induced angiogenesis in rat skeletal muscle. Am. J. Physiol. Heart Circ. Physiol..

[10] Hsu SL (2019). Hyperbaric oxygen facilitates the effect of endothelial progenitor cell therapy on improving outcome of rat critical limb ischemia. Am. J. Transl. Res..

[11] Stephen RT (2009). Oxidative stress is fundamental to hyperbaric oxygen therapy. J. Appl. Physiol..

[12] Flantz S, Vincent KA, Feron O, Kelly RA (2005). Innate immunity and angiogenesis. Circ. Res..

[13] Latroche C (2015). Skeletal Muscle Microvasculature: A Highly Dynamic Lifeline. Physiol..

[14] Katina MF, Stephen RT (2014). Hyperbaric Oxygen, Vasculogenic Stem Cells, and Wound Healing. Antioxid. Redox Signal..

[15] Phillips GD (1991). An angiogenic extractfrom skeletal muscle stimulates monocyte and endothelial cell chemotaxis *in vitro*. Proc. Soc. Exp. Biol. Med..

[16] Ciprian, H. *et al*. An overview of protective strategies against ischemia/reperfusion injury: The role of hyperbaric oxygen preconditioning. *Brain and Behavior*. 1–14 (2018) 10.1002/brb3.959 PMC5943756 29761012

[17] Jamieson D, Chance B, Cadenas E, Boveris A (1986). The relation of free radical production to hyperoxia. Annu. Rev. Physiol..

[18] Allen R, Balin A (1989). Oxidative influence on development and differentiation: an overview of a free radical theory of development. Free. Radic. Biol. Med..

[19] Calabrese V (2007). Nitric oxide in the central nervous system: neuroprotection versus neurotoxicity. Nat. Rev. Neurosci..

[20] Maulik N (2002). Redox signaling and angiogenesis. Antioxid. Redox Signal..

[21] Tompach PC, Lew D, Stoll JL (1997). Cell response to hyperbaric oxygen treatment. Int. J. Oral. Maxillofac. Surg..

[22] Semenza GL (2001). HIF-1 and mechanisms of hypoxia sensing. Curr. Opin. Cell Biol..

[23] Ishii Y (1999). Effect of hyperbaric oxygen on procollagen on messenger RNA level and collagen synthesis in the healing of rat tendon laceration. Tissue Eng..

[24] Conconi MT (2003). Effects of hyperbaric oxygen on proliferative and apoptotic activities and reactive oxygen species generation in mouse fibroblast 3T3/J2 cell line. J. Investig. Med..

[25] Pompella A, Visvikis A, Paolicchi A, De Tata V, Casini AF (2003). The changing faces of glutathione, a cellular protagonist. Biochem. Pharmacol..

[26] Stephen RT (2011). Hyperbaric oxygen - its mechanisms and efficacy. Plast. Reconstr. Surg..

[27] Heng MC (2000). Angiogenesis in necrotic ulcers treated with hyperbaric oxygen. Ostomy Wound Manage..

[28] Bennet HM, Kertesz T, Perleth M, Yeung P, Lehm JP (2012). Hyperbaric oxygen for idiopathic sudden sensorineural hearing loss and tinnitus. Cochrane Database Syst. Rev..

[29] Ishii Y (2005). Hyperbaric Oxygen as an Adjuvant for Athletes. Sports Med..

[30] Yoshimasa I (2005). Hyperbaric Oxygen as an Adjuvant for Athletes. Sports Med..

[31] Horie M (2014). Enhancement of satellite cell differentiation and functional recovery in injured skeletal muscle by hyperbaric oxygen treatment. J. Appl. Physiol..

[32] Oyaizu T (2018). Hyperbaric oxygen reduces inflammation, oxygenates injured muscle, and regenerates skeletal muscle via macrophage and satellite cell activation. Sci. Reports..

[33] Kasemkijwattana C (1998). Biologic intervention in muscle healing and regeneration. Sports Med. Arthrosc. Rev..

[34] Evangelos J (2008). Kinetics of Angiopoietin-2 in serum of multi-trauma patients: Correlation with patient severity. Cytokine..

[35] Morikawa S, Ezaki T (2011). Imaging and Quantification of Plasma Leakage from Microvessels by Using Intravital Lectin Injection. Microscopy..

[36] Richrd LB, Melissa AM, Danielle RM, Theo H, Scott RW (2008). Griffonia simplicifolia isolectin B4 identifies a specific subpopulation of angiogenic blood vessels following contusive spinal cord injury in the adult mouse. J. Comp. Neurol..

[37] Yevgeny B, Alessandra BE, Ruth RC, Michael A, David JM (2013). Enhancing microvascular formation and vessel maturation through temporal control over multiple pro-angiogenic and pro-maturation factors. Biomaterials..

[38] Emmeran LM (2017). Redox Control of Skeletal Muscle Regeneration. Antioxid. Redox Signal..

[39] Víteček, J., Lojek, A., Valacchi, G. & Kubala, L. Arginine-Based Inhibitors of Nitric Oxide Synthase: Therapeutic Potential and Challenges. Mediators of Inflammation. 318087, https://www.hindawi.com/journals/mi/2012/318087/ (2012). 10.1155/2012/318087 PMC3441039 22988346

[40] Erdogdu OD, Nathanson D, Sjoholm A, Nystrom T, Zhang Q (2010). Exendin-4 stimulates proliferation of human coronary artery endothelial cells through eNOS-, PKA- and PI3K/Akt-dependent pathways and requires GLP-1 receptor. Mol. Cell. Endocrinology..

[41] Filippin LI (2011). Nitric oxide regulates the repair of injured skeletal muscle. Nitric Oxide..

[42] Gillian G, Francesca D, Thomas G, Maryanne D (2012). Reactive Oxygen Species Regulate Prosurvival ERK1/2 Signaling and bFGF Expression in Gliosis within the Retina. Investig. Ophthalmol. Vis. Science..

[43] Li WQ, Qureshi HY, Liacini A, Dehnade F, Zafarullah M (2004). Transforming growth factor Beta1 induction of tissue inhibitor of metalloproteinases 3 in articular chondrocytes is mediated by reactive oxygen species. Free. Radic. Biology..

[44] Wong WC, Lerner E (2015). Nitric oxide inhibition strategies. Future Sci. OA..

[45] Jan, V., Antonin, L., Giuseppe, V. & Lukas, K. Arginine-Based Inhibitors of Nitric Oxide Synthase: Therapeutic Potential and Challenges. *Mediators of Inflammation*. 1–22 (2012). 10.1155/2012/318087 PMC3441039 22988346

[46] Murohara T (1998). Vascular endothelial growth factor/vascular permeability factor enhances vascular permeability via nitric oxide and prostacyclin. Circulation..

[47] Olukayode O (2019). *In Vitro* and *In Vivo* Antioxidant Properties of Taraxacum officinale in Nω-Nitro-l-Arginine Methyl Ester (L-NAME)-Induced Hypertensive Rats. Antioxid..

[48] Murakami M (2008). The FGF system has a key role in regulating vascular integrity. J. Clin. Invest..

[49] Bottaro DP (1991). Identifi cation of the hepatocyte growth factor receptor as the c-met proto-oncogene product. Science..

[50] Marie PS, Roselyne T, Jacques P (2008). The Angiopoietin-2 Gene of Endothelial Cells is Up-Regulated in Hypoxia by a HIF Binding Site Located in Its First Intron and by the Central Factors GATA-2 and Ets-1. Physiol..

[51] Buckley, C. J. & Jeffrey, S. C. Hyperbaric, Angiogenesis. *Statpearls*. 2018

[52] Dulak J, Jozkowicz A (2003). Regulation of vascular endothelial growth factor synthesis by nitric oxide: facts and controversies. Antioxid. Redox Signal..

[53] Ziche M (1997). Nitric Oxide Promotes Proliferation and Plasminogen Activator Production by Coronary Venular Endothelium Through Endogenous bFGF. Circ. Res..

[54] Narkowicz CK, Vial JH, McCatrtney PW (1993). Hyperbaric oxygen therapy increases free radical levels in the blood of humans. Free. Radic. Res. Commun..

[55] Kami K (1993). Changes of vinculin and extracellular matrix components following blunt trauma to rat skeletal muscle. Med. Sci. Sports Exerc..

[56] Hurme T, Kalimo H (1992). Activation of myogenic precursor cells after muscle injury. Med. Sci. Sports Exerc..

[57] Nicolas NN (1998). Vascular Endothelial Growth Factor Mediates Angiogenic Activity during the Proliferative Phase of Wound Healing. Am. J. Pathol..

[58] Piepmeier EH, Kalns JE (1999). Fibroblast response to rapid decompression and hyperbaric oxygenation. Aviat. Space Env. Med..

[59] Kobayasi T (1996). Surgical treatment for Achilles tendon rupture. Orthopedics Traumatology..

