# Supplementary material for: VEGF and bFGF induction by nitric oxide is associated with hyperbaric oxygen-induced angiogenesis and muscle regeneration
Source: Sci Rep. 2020 Feb 17;10:2744. doi: 10.1038/s41598-020-59615-x (PMC7026099; doi:10.1038/s41598-020-59615-x)
Supplement: Supplementary file 1 — Supplementary information. [file 41598_2020_59615_MOESM1_ESM.docx]

**Supplementary Information**

**Title**

**VEGF and bFGF induction by nitric oxide is associated with hyperbaric oxygen-mediated angiogenesis and muscle regeneration.**

**Authors and affiliations**

Naoki Yamamoto ^a, b^ , Takuya Oyaizu ^b, c^, Mitsuhiro Enomoto ^a^, Masaki Horie ^b^, Masato Yuasa ^a^, Atsushi Okawa ^a^, Kazuyoshi Yagishita ^b^

a Department of Orthopaedic Surgery, Tokyo Medical and Dental University, Bunkyo-ku, Tokyo 113-8519, Japan

b Hyperbaric Medical Center, Medical Hospital, Tokyo Medical and Dental University, Tokyo 113-8519, Japan

c Saiseikai Kawaguchi General Hospital, Kawaguchi-shi, Saitama 332-8558, Japan

**Corresponding Authors**

Takuya Oyaizu, M.D., Ph.D.

Hyperbaric Medical Center and Sports Medicine Clinical Center, Medical Hospital, Tokyo Medical and Dental University

1-5-45, Yushima, Bunkyo-ku, Tokyo 113-8519, Japan

Phone: +81-3-5803-5279, Fax: +81-3-5803-5281

E-mail: oyaizu.orth@tmd.ac.jp


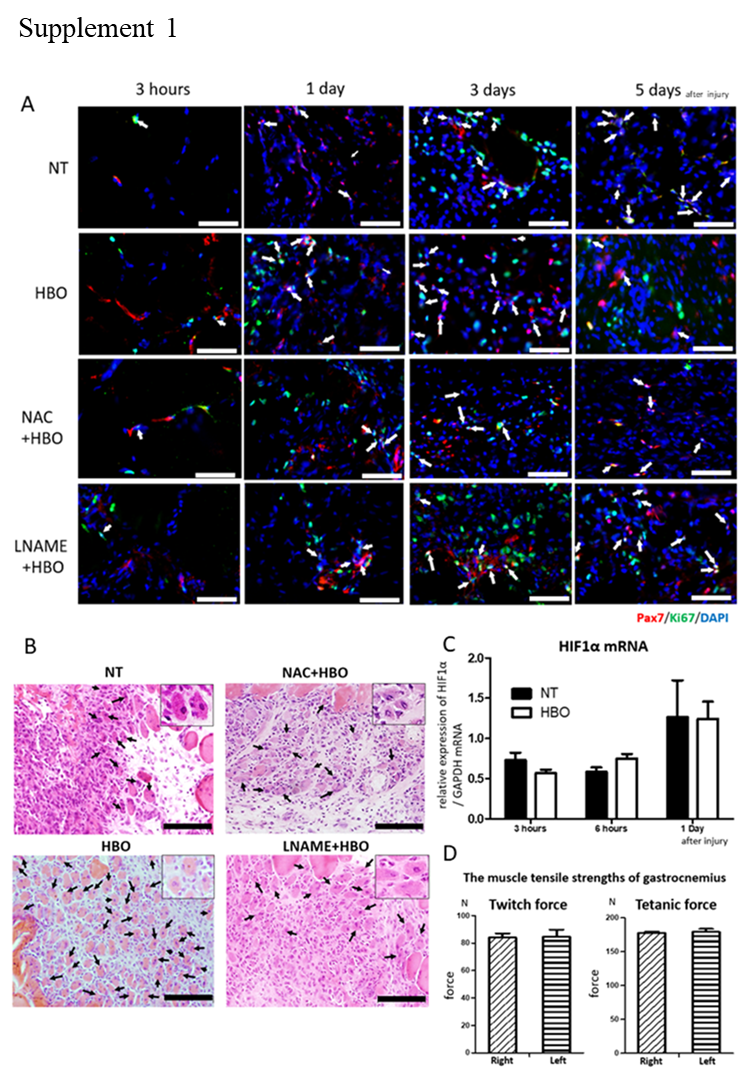


**Figure S1. Effects of NAC and L-NAME inhibition on muscle regeneration.**

(A) Representative image of satellite cells positive for Pax7 (red), Ki67 (green), and DAPI (blue) in injured muscle at 3 hours and 1, 3, and 5 days after injury (arrows). Scale bar: 100 µm. (B) Representative image of regenerating muscle fibers with center nuclei (arrows) in HE-stained sections at 5 days after injury. Scale bar: 100 µm. One of the regenerating muscle fibers is enlarged in the upper right at each image. (C)There was no significant difference in the relative expression of HIF1α mRNA, n = 4, using multiple-way ANOVA followed by Bonferroni post-tests. 3 hours, F (1,6) = 2.692, 6 hours- F (1,6) = 4.682; 1 day, F (1,6) = 0.002. (D) There was no significant difference in the right and left gastrocnemius muscle tensile strength, n = 6, using Welch’s t-test. Data are the mean ± SEM.

**
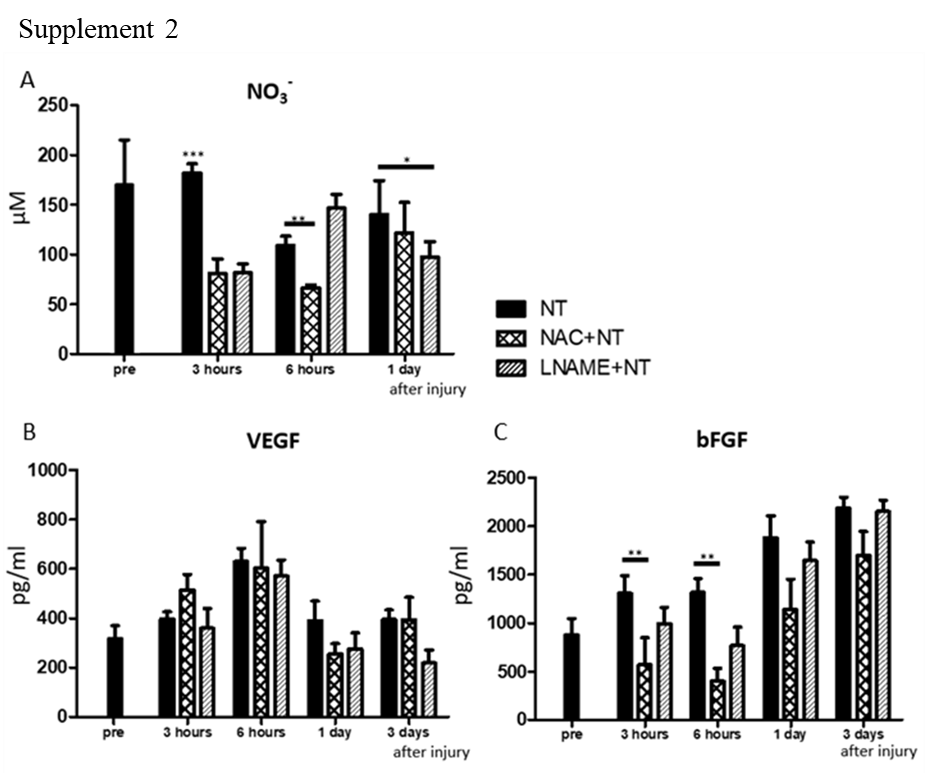
**

**Figure S2. Effects of NAC and L-NAME inhibition in the NT group on NO_3_^-^, VEGF and bFGF.**

Under inhibition by NAC or L-NAME, the amounts of (A) NO_3_^-^ and (C) bFGF were significantly suppressed compared to the NT group; however, there was no significant difference for the amount of (B) VEGF, n = 6, 48 animals total, using multiple-way ANOVA followed by Bonferroni post-tests, (A) 3 hours, F (2,15) = 26.9, p < 0.001; 6 hours, F (2,15) = 19.02, p = 0.004; 1 day, F(2,15) = 2.806, p = 0.034; (B) 3 hours, F (2,15) = 2.482; 6 hours, F (2,15) = 0.291; 1 day, F(2,15) = 0.972; 3 days, F(2,15) = 1.997; (C) 3 hours, F (2,15) = 2.954, p = 0.029; 6 hours, F (2,15) = 8.455, p = 0.027; 1 day, F(2,15) = 2.318, 3 days- F(2,15) = 2.524.

**
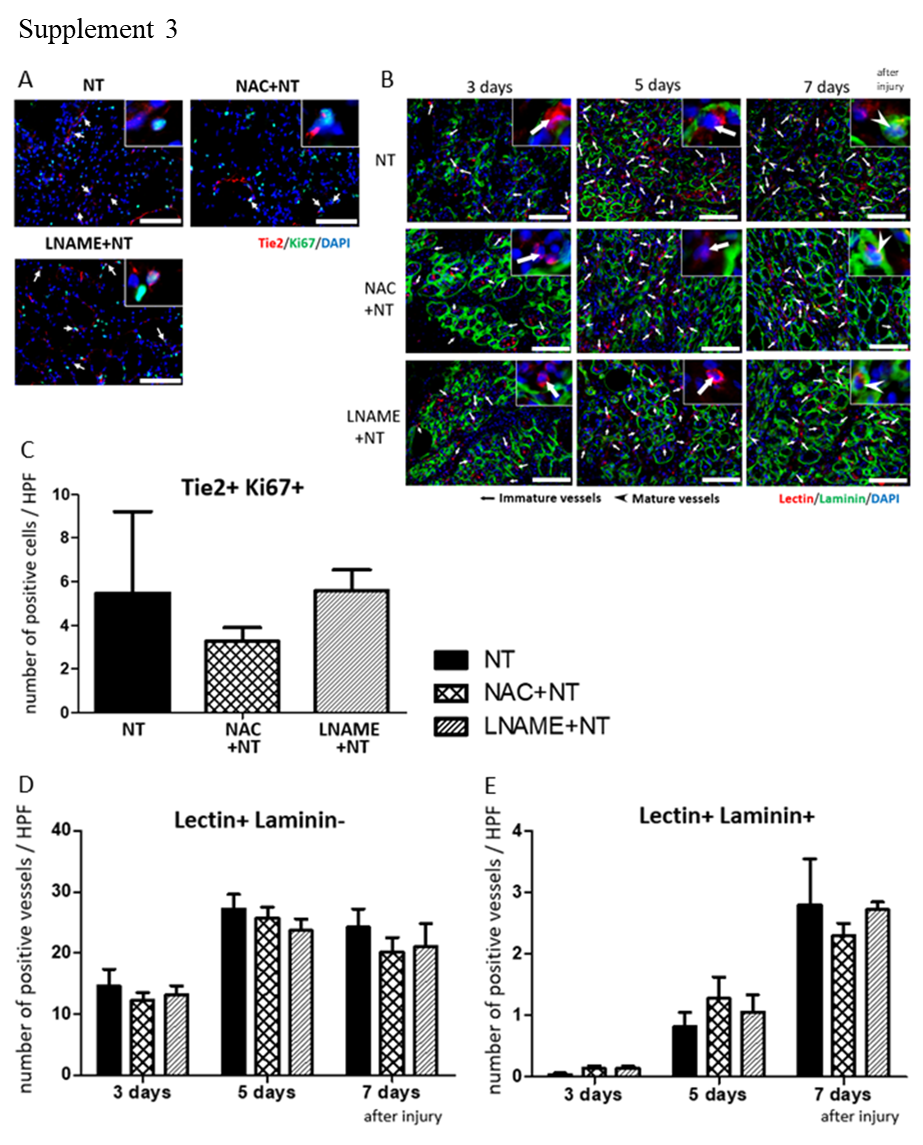
**

**Figure S3. Effects of NAC and L-NAME inhibition in the NT group on angiogenesis.**

(A) Representative images of endothelial cells positive for Tie2 (red), Ki67 (green), and DAPI (blue) in injured muscle (arrows). Scale bar: 100 µm. One of the double-positive cells is enlarged in the upper right of each image. (B) Representative images of immature blood vessels (inset) positive for tomato lectin (red) and DAPI (blue) (arrows), and mature vessels (inset) positive for tomato lectin, laminin (green), and DAPI (arrow heads) in injured muscle. Scale bar: 100 µm. One of the immature or mature vessels is enlarged in the upper right at each image. (C) There was no significant difference in the number of Tie2 and Ki67 double-positive cells, n = 5, total 20 animals, using one-way ANOVA followed by Bonferroni post-tests, F (2,12) = 0.134. Data are the mean ± SEM. (D, E) There was no significant difference in the number of immature and mature vessels, n = 5, using multiple-way ANOVA followed by Bonferroni post-tests. (D) 3 days, F (2,12) = 0.331; 5 days, F (2,12) = 0.715; 7 days, F (2,12) = 0.539; (E) 3 days, F (2,12) = 2.632; 5 days, F (2,12) = 0.638; 7 days, F (2,12) = 0.357.


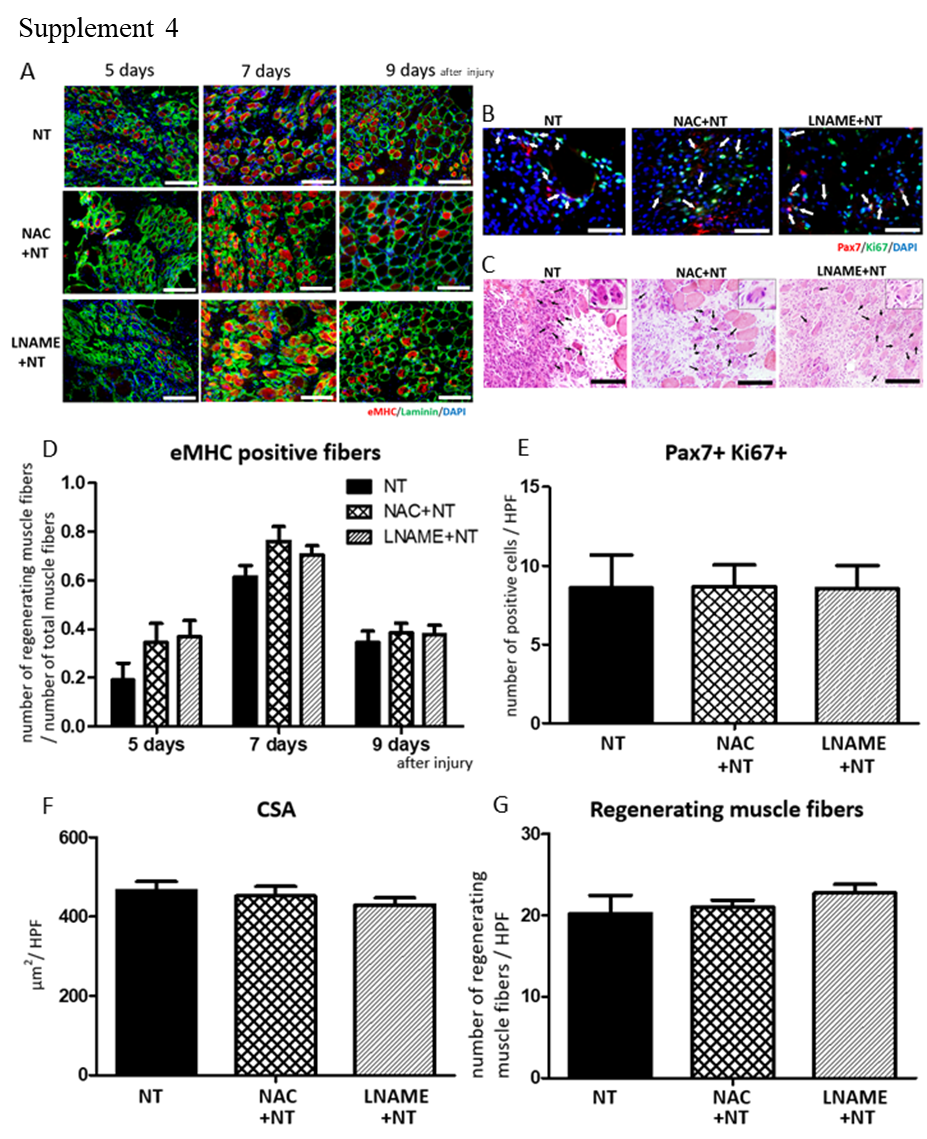


**Figure S4. Effects of NAC and L-NAME inhibition in the NT group on muscle regeneration.**

(A) Representative image of regenerating muscle fibers stained for eMHC and laminin at 5, 7, and 9 days after injury. Scale bar: 100 µm. (B) Representative image of satellite cells positive for Pax7 (red), Ki67 (green), and DAPI (blue) in injured muscle at 3 hours and 1, 3, and 5 days after injury (arrows). Scale bar: 100 µm. (C) Representative image of regenerating muscle fibers with center nuclei (arrows) in HE-stained sections at 5 days after injury. Scale bar: 100 µm. One of the regenerating muscle fibers is enlarged in the upper right in each image. There was no significant difference for (D) the number of eMHC-positive muscle fibers at each time point, n = 6, using two-way ANOVA followed by Bonferroni post-tests; 5 days, F (2,12) = 1.254; 7 days, F (2,12) = 1.588; 9 days, F (2,12) = 0.37. (E) Number of Pax7 and Ki67 double-positive cells at 3 days after injury, n = 6, using one-way ANOVA followed by Bonferroni post-tests, F (2,12) = 0.002. (F)(G) CSA and the number of regenerating muscle fibers stained with H&E at 5 days after injury, n = 6, using one-way ANOVA followed by Bonferroni post-tests, Data are the mean ± SEM. (D) F (2,12) = 0.776, (E) F (2,12) = 0.834.


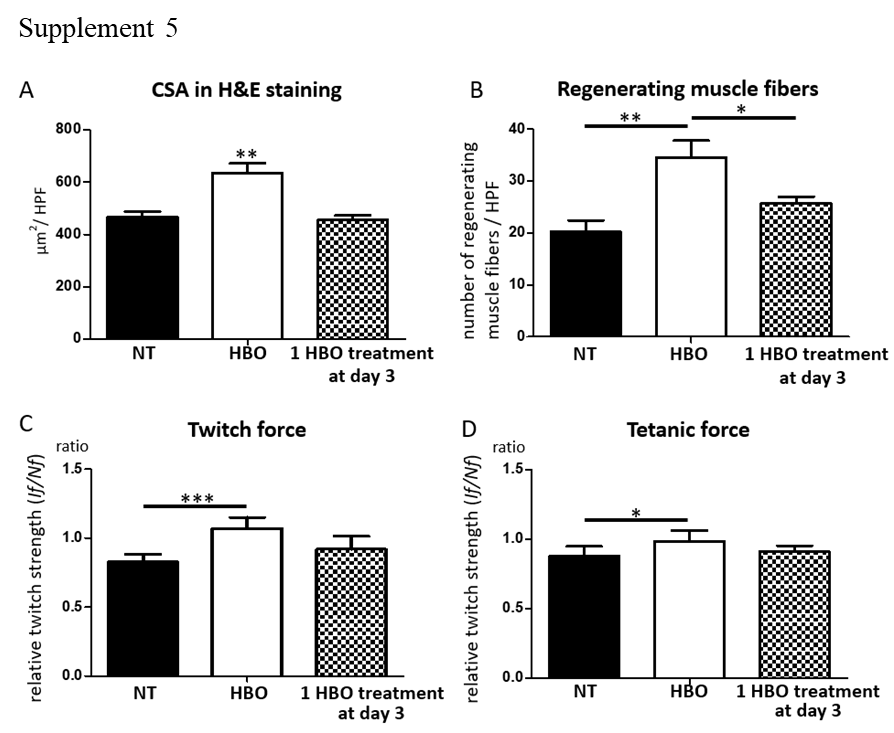


**Figure S5. Effects of a single** **HBO treatment at 3 days after injury on muscle regeneration.**

(A) CSA of regenerating muscle fibers stained with H&E with a single HBO treatment at 3 days (HBO 1T 3D) was not increased, n = 5, using one-way ANOVA followed by Bonferroni post-tests, F (2,12) =13.19, p = 0.002. (B) Number of regenerating muscle fibers in the HBO 1T 3D group was not increased, n = 5, using one-way ANOVA followed by Bonferroni, F (2,12) = 9.206, HBO: p = 0.001, HBO 1T D3: p = 0.022. (C)(D) Twitch and tetanic muscle strength did not recover in the HBO 1T 3D group, n = 6, using one-way ANOVA followed by Bonferroni. Data are the mean ± SEM. (C) F (2,15) = 12.1, p < 0.001 (D) F (2, 15) = 4.621, p = 0.015.
